# Die frühzeitige Behandlung des Erysipels mittels Kompressionstherapie reduziert das C‐reaktive Protein und Symptome – Ergebnisse einer randomisiert kontrollierten Studie

**DOI:** 10.1111/ddg.15829_g

**Published:** 2025-10-23

**Authors:** Sören Dräger, Charlotte Kiehne, Greta Zinser, Birgit Kahle

**Affiliations:** ^1^ Klinik für Dermatologie Allergologie und Venerologie Universitätsklinikum Schleswig‐Holstein (UKSH), Campus Lübeck; ^2^ Klinik für Dermatologie Universitätsklinikum Düsseldorf

**Keywords:** Erysipel, Kompressionstherapie, Medizinisch adaptive Kompressionssystem, cellulitis, compression therapy, medical adaptive compression wraps

## Abstract

**Hintergrund und Ziele:**

Das Erysipel ist eine akute bakterielle Infektion der Haut. Die Erstbehandlung besteht aus einer systemischen Antibiotikatherapie. Später wird eine Kompressionstherapie eingeleitet, um die Ödeme zu lindern. Es gibt eine anhaltende Debatte über den richtigen Zeitpunkt für den Beginn der Kompressionstherapie, da man annimmt, dass ein zu früher Beginn die Infektion verschlimmert.

**Patienten und Methodik:**

Diese Studie wurde als prospektive, randomisiert‐kontrollierte Studie durchgeführt. Patienten, die stationär aufgrund eines Erysipels des Unterschenkels behandelt wurden, wurden rekrutiert und 1:1 randomisiert. Zusätzlich zur Standardtherapie erhielt die Interventionsgruppe einen Tag nach Beginn der Antibiotikabehandlung eine Kompressionstherapie mit medizinischen adaptiven Kompressionssystemen (MAK). Die Serumkonzentration des C‐reaktive Proteins (CRP), der Rückgang der Erythemfläche und die von den Patienten berichteten Symptome wurden erfasst.

**Ergebnisse:**

Insgesamt wurden 34 Patienten in die Analyse einbezogen. Die frühzeitige Anwendung von medizinischen adaptiven Kompressionssystemen führte zu einer Linderung der Symptome, ohne Komplikationen zu verursachen. Bei Patienten mit initialen CRP‐Konzentrationen über 50 mg/dl bei Aufnahme wurde die CRP‐Reduktion beschleunigt.

**Schlussfolgerungen:**

Die Ergebnisse dieser Studie deuten darauf hin, dass die frühzeitige Anwendung eines medizinisch adaptiven Kompressionssystems innerhalb von 24 Stunden nach Beginn der Antibiotikabehandlung die Symptome lindert, die Genesung unterstützt und keine Verschlechterung der Entzündung hervorruft.

## EINLEITUNG

Das Erysipel ist eine akute bakterielle Infektion, die die Haut, das subkutane Gewebe und die dermalen Lymphgefäße betrifft. Sie wird hauptsächlich durch ß‐hämolysierende Streptokokken, in der Regel der Gruppe A, oder *Staphylococcus aureus* verursacht.^1^ In vielen Fällen dienen kleine Defekte der Hautbarriere, die häufig durch eine Pilzinfektion in den Fußzwischenräumen oder eine Ulzeration verursacht werden, als Eintrittspforte für diese Erreger. Das Risiko eines Erysipels wird durch eine chronische Veneninsuffizienz, Lymphödeme, Tinea pedis und andere Faktoren erhöht.^2^ Die Diagnose des Erysipels basiert auf ihrem charakteristischen klinischen Erscheinungsbild, aus typischerweise asymmetrischen, gut definierten Erythemen, lokaler Überwärmung und Schwellungen. In vielen Fällen tritt zusätzlich Fieber auf. Die Unterschenkel, Arme und der Rumpf sind die am häufigsten betroffenen Körperregionen.^3^ Zu den Laborbefunden gehören unspezifisch erhöhte Entzündungsmarker wie eine Leukozytose und erhöhte Serumkonzentrationen des C‐reaktiven Proteins (CRP). Ein direkter Erregernachweis gelingt in der Regel nicht und spielt daher diagnostisch eine untergeordnete Rolle. Komplizierend können lokale Erosionen, Blasen oder hämorrhagische Läsionen auftreten.^4^ Die Bakteriämie oder Sepsis stellen potenziell lebensbedrohliche Komplikationen dar. Eine häufige Sekundärkomplikation des Erysipels ist das Lymphödem, das wiederum das Risiko eines erneuten Erysipels erhöht.^5^, ^6^ Die Therapie des Erysipels erfolgt durch die Gabe von Antibiotika, wobei in Deutschland Penicillin als erste Wahl empfohlen wird.^7^ Zu den unterstützenden Maßnahmen gehören körperliche Schonung, kühlende Kompressen, Schmerztherapie und die Sanierung der Eintrittspforte.

Zusätzlich kann eine Kompressionstherapie durchgeführt werden. Sie fördert die Durchblutung der Haut, beschleunigt den Lymphabfluss, mildert Ödeme und wird häufig bei venösen und lymphatischen Erkrankungen eingesetzt.^8^, ^9^, ^10^ Vor Therapiebeginn sollte routinemäßig eine klinische Untersuchung der peripheren arteriellen Durchblutung erfolgen – zum Beispiel durch Palpation der Arteria dorsalis pedis oder der Arteria tibialis posterior –, insbesondere bei entsprechender Risikokonstellation (wie periphere arterielle Verschlusskrankheit, Diabetes mellitus oder periphere Neuropathie). Kontraindikationen für eine Kompressionstherapie sind ein Knöchel‐Arm‐Index unter 0,5, ein am Knöchel gemessener systolischer Blutdruck unter 60 mmHg, eine septische Phlebitis, eine Phlegmasia coerulea dolens oder eine dekompensierte Herzinsuffizienz.^10^ Der optimale Zeitpunkt für den Beginn der Kompressionstherapie ist umstritten.^11^ Früher wurde erst mehrere Tage nach Initiierung der Antibiotikabehandlung mit einer Kompressionstherapie begonnen, da die Sorge bestand, dass die Kompression während der akuten Entzündungsphase die Infektion verschlimmern und möglicherweise eine Sepsis auslösen könnte. Bisherige Studien konnten jedoch zeigen, dass die Kompressionstherapie den Verlauf von bakteriellen Hautinfektionen nicht nachteilig beeinflusst. Im Gegenteil, die Kompressionstherapie scheint die Abheilung zu fördern.^11^ Die Sekundärprävention von rezidivierenden Erysipelen mittels Kompressionstherapie ist gut etabliert und kosteneffektiv.^12^, ^13^ Es gibt verschiedene Kompressionssysteme, einschließlich medizinisch adaptiver Kompressionssysteme (MAK).

Ein Vorteil der MAK ist ihre Fähigkeit, sich insbesondere in der Abschwellungsphase dynamisch an veränderte Umfangmaße anzupassen. Darüber hinaus sind die einfache Handhabung und die Möglichkeit der selbstständigen Anwendung durch den Patienten vorteilhaft, was die Compliance und die regelmäßige Anwendung unterstützt.

Zusammenfassend gibt es derzeit nicht genügend Studien, die den optimalen Zeitpunkt für den Beginn einer Kompressionstherapie untersucht haben. Deshalb wurde in dieser Studie untersucht, ob die unverzügliche Einleitung der Kompressionstherapie, das heißt die Anwendung von MAK innerhalb von 24 Stunden nach Beginn der Antibiotikatherapie, zu einer beschleunigten Reduktion der CRP‐Werte, einer Verringerung der Erythemfläche und einer Verbesserung der Symptome führt.

## PATIENTEN UND METHODIK

**ABBILDUNG 1:**
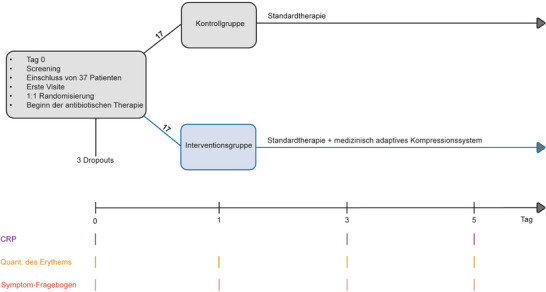
Schematische Darstellung des Studienablaufs. Am Aufnahmetag (Tag 0) wurde die antibiotische Behandlung eingeleitet. Anschließend erfolgte die Aufklärung über die Studie und der Einschluss nach Einwilligung. Die initiale Visite fand am selben Tag statt (C‐reaktives Protein [CRP], Erythem, Symptomfragebogen). Sowohl die Kontroll‐ als auch die Interventionsgruppe erhielten die Standardbehandlung. Am Tag 1 wurde in der Interventionsgruppe zusätzlich ein individuell angepasstes medizinisch adaptives Kompressionssystem angelegt und die Patienten in dessen Anwendung geschult. Die Quantifizierung der Erythemfläche sowie der Symptomfragebogen wurden an den Tagen 1, 3 und 5 erhoben; die CRP‐Werte wurden an den Tagen 3 und 5 erneut bestimmt. Nach Tag 5 wurde die Behandlung fortgeführt, jedoch im Rahmen dieser Studie nicht weiter dokumentiert.

Diese Studie wurde als prospektive, randomisierte, kontrollierte Studie durchgeführt, die von der lokalen Ethikkommission der Universität Lübeck (AZ 21–043) genehmigt wurde und mit der Deklaration von Helsinki aus dem Jahr 1975 konform ist. Nach informierter Zustimmung wurden Patienten im Alter von 18 Jahren und älter, die zur Behandlung eines Erysipels des Unterschenkels stationär aufgenommen wurden, in die Studie eingeschlossen. Alle Patienten wurden zwischen Oktober 2021 und März 2024 in der Klinik für Dermatologie, Allergologie und Venerologie am Universitätsklinikum Schleswig‐Holstein, Campus Lübeck, behandelt. Ausschlusskriterien waren das Vorliegen einer Phlegmone, eine dekompensierte Herzinsuffizienz und die Unfähigkeit, das MAK selbstständig anzuwenden. Die Teilnehmer wurden nach dem Zufallsprinzip im Verhältnis 1:1 in zwei Gruppen randomisiert: Die Kontrollgruppe erhielt die Standardtherapie mit intravenösen Antibiotika, antiseptischen Kompressen und eingeschränkter Bettruhe. Die MAK‐Gruppe erhielt dieselbe Standardtherapie wie die Kontrollgruppe und zusätzlich die Kompressionstherapie mit MAK, welche 24 Stunden nach Beginn der Antibiotikabehandlung eingeleitet wurde (Abbildung 1). Ein 24‐Stunden‐Intervall wurde als definiertes Zeitfenster gewählt, um den Wirkungseintritt der antibiotischen Therapie vor Beginn der Kompressionstherapie abzuwarten. In dieser Gruppe wurden zweimal täglich 30 Minuten lang antiseptische Wickel angelegt, bevor die MAK angewendet wurden. Die Randomisierung erfolgte durch fortlaufend nummerierte versiegelte Umschläge, die erst nach schriftlicher Studieneinwilligung der Patienten geöffnet wurden. Die Randomisierungssequenz wurde vom Institut für Medizinische Biometrie und Statistik der Universität zu Lübeck erstellt. Die Behandlung wurde bis zur Entlassung fortgeführt. Die medizinische Betreuung und Entscheidungsfindung erfolgte durch Ärztinnen und Ärzte, die nicht an der Durchführung der Studie beteiligt waren.

Der primäre Endpunkt der Studie war die Abnahme der CRP‐Werte, die an den Tagen 0, 3 und 5 gemessen wurden. Sekundäre Endpunkte waren die Veränderung der Erythemfläche gegenüber dem Ausgangswert und die berichteten Symptome. Die Fläche des Erythems wurde an den Tagen 0, 1, 3 und 5 quantifiziert, indem eine definierte transparente Folie auf die betroffene Stelle gelegt und die Grenzen des Erythems auf die Folie übertragen wurde. Die Folie wurde dann entsprechend zugeschnitten und gewogen. Die absoluten Werte in Gramm wurden notiert und die relative Veränderung gegenüber dem Ausgangswert berechnet. Die Symptome wurden an den Tagen 0, 1, 3 und 5 anhand eines modifizierten Symptomfragebogens auf Papier bewertet, der sieben Items umfasste, die jeweils auf einer Skala von 0 bis 10 bewertet wurden. Die bewerteten Items waren die folgenden: *(1.)* Wie stark fühlen Sie sich durch Ihre Krankheit beeinträchtigt? *(2.)* Wie stark sind Ihre Schmerzen? *(3.)* Wie unangenehm ist die Schwellung am Bein? *(4.)* Wie unangenehm ist die Schwellung der Füße? *(5.)* Haben Sie Schmerzen beim Gehen? *(6.)* Haben Sie Schmerzen beim Sitzen? *(7.)* Wie unangenehm ist die Kompression? Item 7 galt ausschließlich für die MAK‐Gruppe und wurde daher separat ausgewertet. Darüber hinaus wurden Daten zur Medikation, Anamnese und Aufenthaltsdauer aus den Krankenakten entnommen.

Die Berechnung des Stichprobenumfangs erfolgte mit Alpha 5%, Power 80% und einer Behandlungseffektgröße von 20%. Die statistische Auswertung erfolgte mit Graph Pad Prism Version 10.0 (GraphPad Software, Inc., USA), R‐Studio (Posit Software, PBC, USA), Microsoft Excel V. 16.87 (Microsoft, USA). Ein p‐Wert < 0,05 wurde als statistisch signifikant gewertet. Die Signifikanzanalyse erfolgte mittels Student's‐*t*‐Test. Eine Subgruppenanalyse wurde bei Patienten mit einer initialen CRP‐Serumkonzentration > 50 mg/dl bei Aufnahme durchgeführt.

## ERGEBNISSE

### Epidemiologie der Studienpopulation

An der Studie nahmen 37 Patienten teil. Drei Patienten wurden von der Analyse ausgeschlossen: zwei (je einer pro Gruppe), da keine weiteren Daten über Tag 1 hinaus erhoben wurden, und ein weiterer Patient, da er die Kompressionstherapie nicht tolerierte und die Studie nach Tag 1 abbrach. Tabelle 1 fasst die Charakteristika der verbleibenden 34 Patienten zusammen, die sich zu gleichen Teilen auf die Kontrollgruppe (n  =  17) und die Interventionsgruppe (n  =  17) verteilen. In der Kontrollgruppe waren 35,3% der Patienten weiblich und 64,7% männlich, während es in der Interventionsgruppe 29,4% Frauen und 70,6% Männer waren. Das Durchschnittsalter betrug 59,0 Jahre (± 23,8) in der Kontrollgruppe und 57,2 Jahre (± 17,1) in der Interventionsgruppe, mit einem Gesamtdurchschnitt von 58,1 Jahren (± 20,4). Der durchschnittliche BMI lag bei 28,98 kg/m^2^ (Kontrollgruppe) beziehungsweise 33,30 kg/m^2^ (Interventionsgruppe) und war damit in der Interventionsgruppe höher.

**TABELLE 1:** Epidemiologie der Studienpopulation. Alter und Geschlecht waren in beiden Gruppen vergleichbar, der Body‐Mass‐Index (BMI) war in der Interventionsgruppe höher. Die Komorbiditäten wurden nach Organsystemen kategorisiert, darunter Herz‐Kreislauf‐, Nieren‐, Autoimmun‐ und Atemwegserkrankungen sowie metabolische, neurologische und psychiatrische Erkrankungen. Zusätzlich wurden Krebserkrankungen und frühere Erysipel‐Episoden erfasst. Die Häufigkeiten sind als absolute Zahlen und entsprechende relative Prozentwerte dargestellt.

Gruppe	Kontrolle		Intervention (MAK)		Gesamt	
	*Anzahl*	*Prozent*	*Anzahl*	*Prozent*	*Anzahl*	*Prozent*
Patienten pro Gruppe	17	50,00%	17	50,00%	34	100,00%
Weiblich	6	35,29%	5	29,41%	11	32,35%
Männlich	11	64,71%	12	70,59%	23	67,65%
Alter (Mittelwert)	59,00		57,16		58,08	
Alter (Standardabweichung)	23,82		17,10		20,44	
BMI (kg/m^2^)	28,98		33,30		31,14	
Kardiovaskuläre Erkrankungen	13	76,47%	9	52,94%	22	64,71%
Nierenerkrankungen	2	11,76%	3	17,65%	5	14,71%
Lungenerkrankungen	2	11,76%	4	23,53%	6	17,65%
Metabolische Erkrankungen	3	17,65%	3	17,65%	6	17,65%
Diabetes mellitus	5	29,41%	2	11,76%	7	20,59%
Autoimmunerkrankungen	2	11,76%	1	5,88%	3	8,82%
Krebserkrankungen	4	23,53%	2	11,76%	6	17,65%
Neurologische Erkrankungen	2	11,76%	0	0,00%	2	5,88%
Psychiatrische Erkrankungen	1	5,88%	1	5,88%	2	5,88%
Vorheriges Erysipel (jede Lokalisation)	1	5,88%	6	35,29%	7	20,59%

*Abk*.: MAK, medizinisch adaptives Kompressionssystem.

Anamnestisch waren Herz‐Kreislauf‐Erkrankungen am häufigsten vertreten – bei 76,5% der Patienten in der Kontrollgruppe und 52,9% in der Interventionsgruppe. Nierenerkrankungen wurden von 11,8% beziehungsweise 17,7% der Patienten angegeben, Atemwegserkrankungen traten bei 11,8% in der Kontroll‐ und bei 23,5% in der Interventionsgruppe auf. Stoffwechselstörungen waren bei 17,7% der Patienten beider Gruppen zu beobachten. Diabetes mellitus wurde in der Kontrollgruppe häufiger berichtet (29,4%) als in der Interventionsgruppe (11,8%). Weitere Erkrankungen waren Malignome (23,5% vs. 11,8%), Autoimmunerkrankungen (11,8% vs. 5,9%) und neurologische Störungen (11,8% in der Kontrollgruppe, keine in der Interventionsgruppe). Psychiatrische Erkrankungen waren in beiden Gruppen gleich häufig vertreten (5,9%). Ein früheres Erysipel wurde häufiger in der Interventionsgruppe dokumentiert (35,3% vs. 5,9%).

### Die frühzeitige Anwendung von MAK lindert Symptome

**ABBILDUNG 2:**
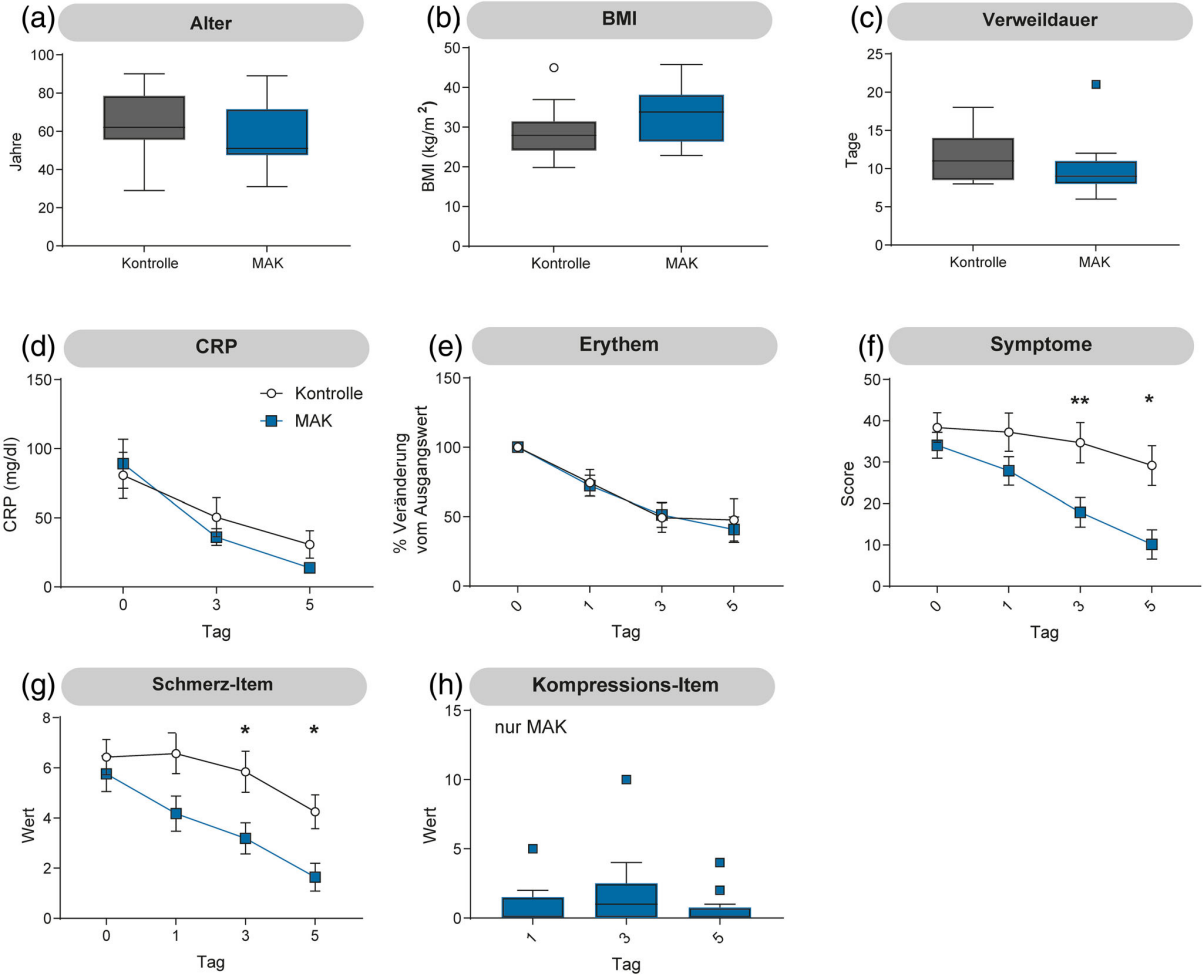
Anwendung eines medizinisch adaptiven Kompressionssystems (MAK) innerhalb von 24 Stunden lindert die Symptome. (a) Alter, (b) Body‐Mass‐Index (BMI), (c) Krankenhausverweildauer, (d) Konzentration des C‐reaktiven Proteins (CRP) und (e) Veränderung der Erythemfläche gegenüber dem Ausgangswert waren zwischen der Interventionsgruppe (MAK) und der Kontrollgruppe vergleichbar. (f) Der Symptomscore, basierend auf einem Fragebogen mit sechs Items, war in der Interventionsgruppe ab Tag 3 signifikant reduziert. (g) Auch das Schmerz‐Item zeigte eine vergleichbare Reduktion. (h) Ein Patient berichtete an Tag 3 über kompressionsbedingte Schmerzen; bei allen anderen Patienten blieb der entsprechende Wert über den gesamten Studienverlauf niedrig. (a–c): Tukey‐Boxplot; (d–h): Mittelwert und Standardfehler des Mittelwerts [SEM]; n  =  17; (a–g): *t*‐Test; *p < 0,05, *p* < 0,005).

Patienten, die innerhalb von 24 Stunden nach Beginn der Antibiotikatherapie mit MAK behandelt wurden, wiesen einen geringeren Symptomwert auf (Abbildung 2f). Beide Gruppen begannen mit einem Symptomwert von 38,4 bzw. 34,1 für die Kontroll‐ und die Behandlungsgruppe. An Tag 3 berichteten die Patienten in der Kontrollgruppe einen mittleren Symptomwert von 34,7, während die MAK‐Behandlung zu einem reduzierten Symptomwert von 17,9 führte. Dieser Unterschied wurde auch an Tag 5 festgestellt, mit einem Symptomwert von 29,2 (Kontrolle) und 10,1 (MAK). Dieser Unterschied wurde auch bei der separaten Betrachtung des Schmerz‐Items des Fragebogens beobachtet (Abbildung 2g).

**ABBILDUNG 3:**
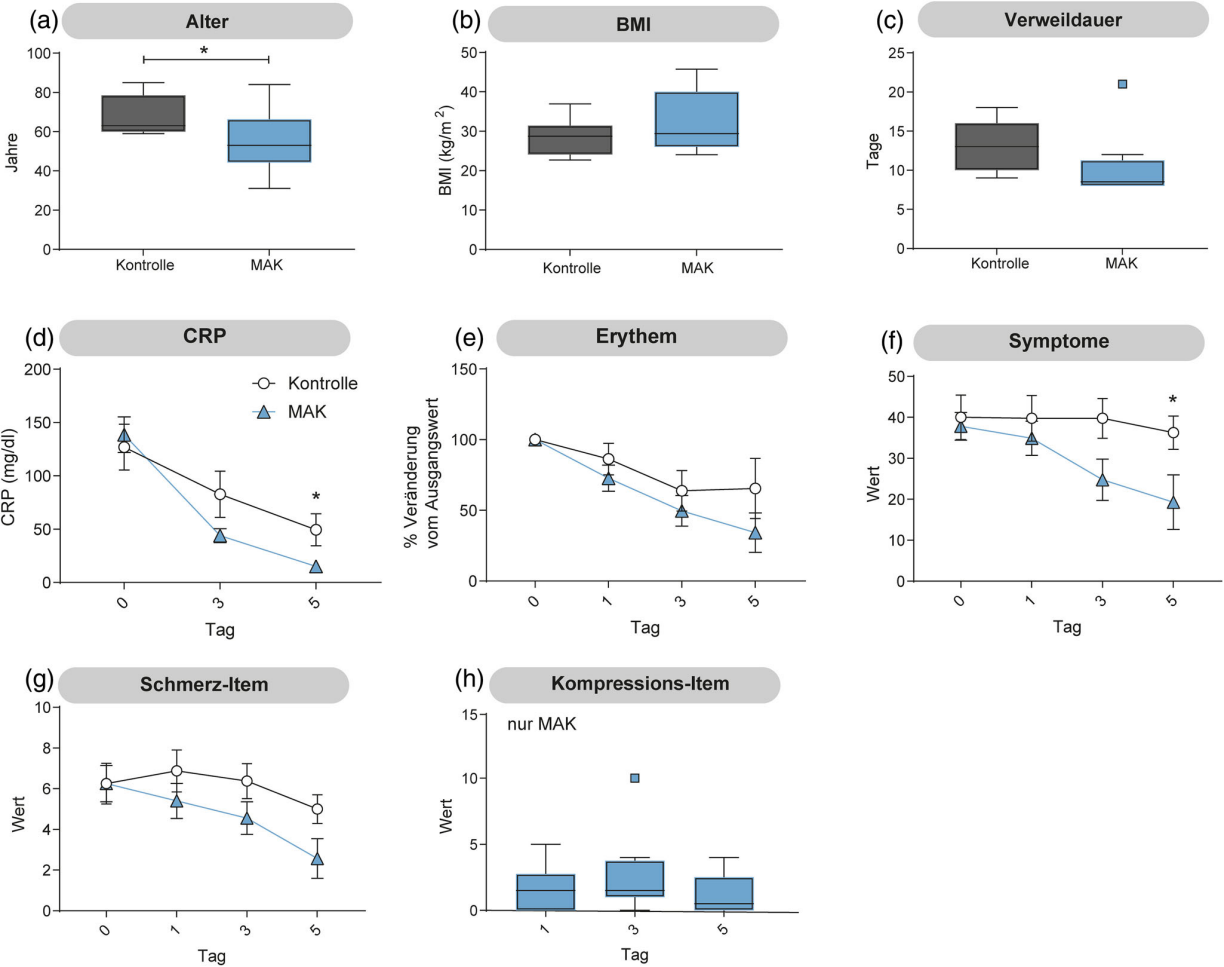
Subanalyse von Patienten mit einer initialen CRP‐Serumkonzentration > 50 mg/dl, bei denen eine beschleunigte CRP‐Reduktion bis Tag 5 beobachtet wurde. (a) Das Alter war in der mit einem medizinisch adaptiven Kompressionssystem (MAK) behandelten Gruppe signifikant niedriger. (b) Der Body‐Mass‐Index (BMI) und (c) die Krankenhausverweildauer waren zwischen beiden Gruppen vergleichbar. (d) Die CRP‐Serumkonzentration war in der Interventionsgruppe an Tag 5 signifikant niedriger. (e) Die Veränderung der Erythemfläche gegenüber dem Ausgangswert war in beiden Gruppen vergleichbar. (f) Der auf dem Fragebogen basierende Symptomscore war in der Interventionsgruppe an Tag 5 signifikant reduziert, (g) während beim Schmerz‐Item kein Unterschied festgestellt wurde. (h) Mit Ausnahme eines Patienten blieben kompressionsbezogene Beschwerden über den gesamten Studienzeitraum hinweg gering. (a–c): Tukey‐Boxplot; (d–h): Mittelwert ± SEM; n  =  9; (a–g): *t*‐Test; *p < 0,05, *p* < 0,005).

Es zeigte sich kein signifikanter Unterschied in der Krankenhausverweildauer zwischen den Gruppen. Die durchschnittliche Krankenhausverweildauer betrug 11,4 Tage in der Kontrollgruppe und 9,95 Tage in der MAK‐Gruppe (Abbildung 2c). In der MAK‐Gruppe trat jedoch ein Ausreißer auf (21 Tage), bedingt durch ein DRESS‐Syndrom (drug reaction with eosinophilia and systemic symptoms) am Ende der Behandlung. Wird dieser Ausreißer aus der Analyse ausgeschlossen, ergibt sich eine leicht veränderte mittlere Krankenhausverweildauer von 9,3 Tagen in der MAK‐Gruppe, und der Unterschied wird statistisch signifikant (*t*‐Test, p  =  0,024). Ein Patient berichtete über starke Beschwerden aufgrund der Kompressionstherapie (Abbildung 2h). Ein Einfluss der MAK‐Behandlung auf die CRP‐Werte (Abbildung 2d) oder die Veränderung der Erythemfläche (Abbildung 2e) konnte nicht festgestellt werden. Auch die Notwendigkeit einer Zweit‐ oder Drittlinien‐Antibiotikatherapie war in der MAK‐Gruppe nicht erhöht (ergänzende Online‐Abbildung 1a). Die eingesetzte Schmerzmedikation war in beiden Gruppen vergleichbar (ergänzende Online‐Abbildung 1b).

### Die Subanalyse der Patienten mit initialem CRP > 50 mg/dl zeigte eine beschleunigte CRP‐Reduktion an Tag 5

Da unsere Einschlusskriterien keine minimale initiale CRP‐Serumkonzentration vorgaben, wiesen die Ausgangswerte eine große Varianz auf. Daher wurde eine Subgruppenanalyse durchgeführt, die nur Patienten mit einem initialen CRP > 50 mg/dl einbezog; dies traf auf jeweils neun Patienten pro Gruppe zu (Abbildung 3).

Unerwartet war das Durchschnittsalter in der Kontrollgruppe signifikant höher als in der MAK‐Gruppe (68,0 vs. 54,4 Jahre; Abbildung 3a). Der BMI war zwischen den Gruppen vergleichbar (Abbildung 3b). Der Trend zur verkürzten Krankenhausverweildauer in der MAK‐Gruppe blieb bestehen und hätte erneut statistische Signifikanz erreicht, wenn der Ausreißer aus der Analyse ausgeschlossen worden wäre (Abbildung 3c). Da für die Subgruppenanalyse eine initiale CRP‐Serumkonzentration von > 50 mg/dl erforderlich war, lagen die mittleren Ausgangswerte in dieser Analyse höher als in der Gesamtpopulation: In der Kontrollgruppe stieg der Mittelwert von 80,6 auf 126,9 mg/dl, in der MAK‐Gruppe von 89,0 auf 138,5 mg/dl (Abbildung 2d. Interessanterweise sanken die CRP‐Serumkonzentrationen in dieser Subanalyse in der MAK‐Gruppe bis Tag 5 schneller. In der Kontrollgruppe blieb die CRP‐Serumkonzentration an Tag 5 mit einem Mittelwert von 49,3 mg/dl relativ hoch, während sie in der MAK‐Gruppe auf 15,0 mg/dl sank. An Tag 5 bestand weiterhin ein signifikanter Unterschied im Symptomwert zwischen den Gruppen; der zuvor beobachtete Unterschied im Schmerz‐Item war hingegen nicht mehr nachweisbar (Abbildung 3e,f).

### Keine Hinweise auf Bakteriämie oder Sepsis unter MAK‐Therapie

Wie bereits erwähnt, entwickelte ein Patient der MAK‐Gruppe im Verlauf der Behandlung ein DRESS‐Syndrom, was eine Verlängerung der Krankenhausverweildauer erforderlich machte. In der Kontrollgruppe kam es bei einem Patienten zu einem Arzneimittelexanthem sowie zu einer Abszessbildung unterhalb des Großzehengrundgelenks, die chirurgisch behandelt wurde. Ein weiterer Patient, der zunächst mit MAK behandelt wurde, brach die Studie an Tag 2 ab. Am siebten Tag entwickelte er eine Phlegmone, die eine Eskalation der antibiotischen Therapie mit Piperacillin/Tazobactam erforderte.

## DISKUSSION

Diese Studie untersuchte, ob die frühzeitige Einleitung einer Kompressionstherapie – der Einsatz von MAK innerhalb von 24 Stunden nach Beginn der Antibiotikabehandlung – die Reduktion der CRP‐Werte beschleunigt, die Erythemfläche verkleinert und die Symptome verringert. Zu diesem Zweck rekrutierten wir insgesamt 37 Patienten in zwei Kohorten, eine Interventionsgruppe (Standardbehandlung + MAK) und eine Kontrollgruppe (nur Standardbehandlung). MAK wurden aufgrund ihrer Vorteile eingesetzt: Sie lassen sich während der Behandlung an den abnehmenden Umfang des Unterschenkels anpassen, sind einfach an‐ und auszuziehen und ermöglichen den Patienten, die Therapie eigenständig durchzuführen. Diese Eigenschaften sind vor allem in der akuten Entzündungsphase des Erysipels entscheidend, in der Schmerzen, Unwohlsein und Beinschwellungen die Hauptsymptome sind. Wir untersuchten daher die Auswirkungen der MAK‐Therapie auf den Symptomscore, der bei Patienten mit MAK signifikant niedriger ausfiel. Besonders der Schmerz‐Score war reduziert, was möglicherweise zu einer verbesserten Compliance beiträgt. Die Anwendung der MAK‐Therapie wurde von einem Patienten nicht gut vertragen, was sich in einer hohen Punktzahl im Kompressionselement widerspiegelte. Zusammenfassend ist die MAK‐Behandlung eine praktikable und gut verträgliche Option zur Behandlung des akuten Erysipels bei Patienten ohne Kontraindikationen für eine Kompressionstherapie. Vor Beginn sollte eine klinische Untersuchung der peripheren arteriellen Durchblutung durchgeführt werden.

Insgesamt beobachteten wir eine vergleichbare Senkung der CRP‐Serumkonzentrationen. Die anfänglich CRP‐Serumkonzentration variierten jedoch stark, da sie in unseren Einschlusskriterien nicht definiert waren. Daher führten wir eine Subgruppenanalyse durch, die nur Patienten mit einer initialen CRP‐Serumkonzentration von > 50 mg/dl einschloss. Diese Subanalyse ergab, dass die MAK‐Gruppe an Tag 5 niedrigere CRP‐Werte aufwies. Da das Ziel dieser Studie darin bestand, die Wirkung von MAK in der Akutphase zu bewerten, wurden in unserem Studiendesign nur Daten bis zum Tag 5 erfasst. Die Wirkung der MAK könnte zu späteren Zeitpunkten noch ausgeprägter gewesen sein. Die in unserer Studie erzielten Werte sind mit denen von Eder et al. vergleichbar, die eine mittlere CRP‐Serumkonzentration von 124,00 mg/dl und von 30,89 mg/dl bei Entlassung beobachteten. Allerdings beinhaltete die Studie von Eder et al. keine Kontrollgruppe.^11^

Hinsichtlich der Komplikationen bei der Anwendung von MAK im Rahmen eines akuten Erysipels konnten keine Unterschiede zwischen den Gruppen festgestellt werden. Die Vorstellung, dass Kompressionstherapie in der Akutphase den Zustand verschlechtern oder eine Sepsis auslösen könnte, ist weit verbreitet. In unserer Studie trat bei keinem Patienten Komplikationen wie Bakteriämie oder Sepsis auf, was mit früheren Ergebnissen übereinstimmt.^11^ Aufgrund der geringen Stichprobengröße sind jedoch größere Kohortenstudien erforderlich, um diese Ergebnisse zu bestätigen.

Unsere Daten deuten darauf hin, dass die unverzügliche Anwendung von medizinisch adaptiven Kompressionssystemen 24 Stunden nach Beginn der Antibiotikabehandlung die Symptome lindert, die Genesung unterstützt und keine Verschlechterung der Entzündung hervorruft.

### Limitationen

Unsere Daten beruhen auf einer Stichprobengröße von 34 Patienten, was die Verallgemeinerung der Ergebnisse einschränkt. Außerdem ist die Studie durch ihre prospektiv‐kontrollierte Datenerhebung ohne Folgeuntersuchung auf den beobachteten Zeitraum limitiert. Da die Daten ausschließlich während des stationären Aufenthalts erfasst wurden, wurden nur primäre Komplikationen berücksichtigt. Folgeereignisse sowie mittel‐ und langfristige Folgen, die im weiteren Verlauf der Behandlung aufgetreten sein könnten, wurden daher nicht berücksichtigt. Dennoch stimmen die erhobenen Daten im Allgemeinen mit der aktuellen Literatur überein. Die Ergebnisse sollten jedoch in Studien mit größeren Stichprobengrößen und längeren Nachbeobachtungszeiträumen weiter untersucht werden.

## DANKSAGUNG

Die Autoren danken der Julius Zorn GmbH (Aichach, Deutschland) für die Bereitstellung der medizinischen adaptiven Kompressionssysteme, dem Institut für Medizinische Biometrie und Statistik (Universität Lübeck, Deutschland) für die Berechnung des Stichprobenumfangs und die Erstellung der Randomisierungssequenz sowie allen Mitarbeiterinnen und Mitarbeitern der Klinik für Dermatologie, Allergologie und Venerologie für ihr Engagement und ihre Fürsorge bei der Behandlung der Patienten.

Open access Veröffentlichung ermöglicht und organisiert durch Projekt DEAL.

## INTERESSENKONFLIKT

G.Z. erhielt eine Forschungsförderung der Julius Zorn GmbH. Alle übrigen Autoren geben an, keine Interessenkonflikte zu haben.

## Supporting information



Supplementary information
