# Supplementary figures and images for: Die frühzeitige Behandlung des Erysipels mittels Kompressionstherapie reduziert das C‐reaktive Protein und Symptome – Ergebnisse einer randomisiert kontrollierten Studie
Source: J Dtsch Dermatol Ges. 2025 Oct 23;23(10):1274–81. [Article in German] doi: 10.1111/ddg.15829_g (PMC12548309; doi:10.1111/ddg.15829_g)

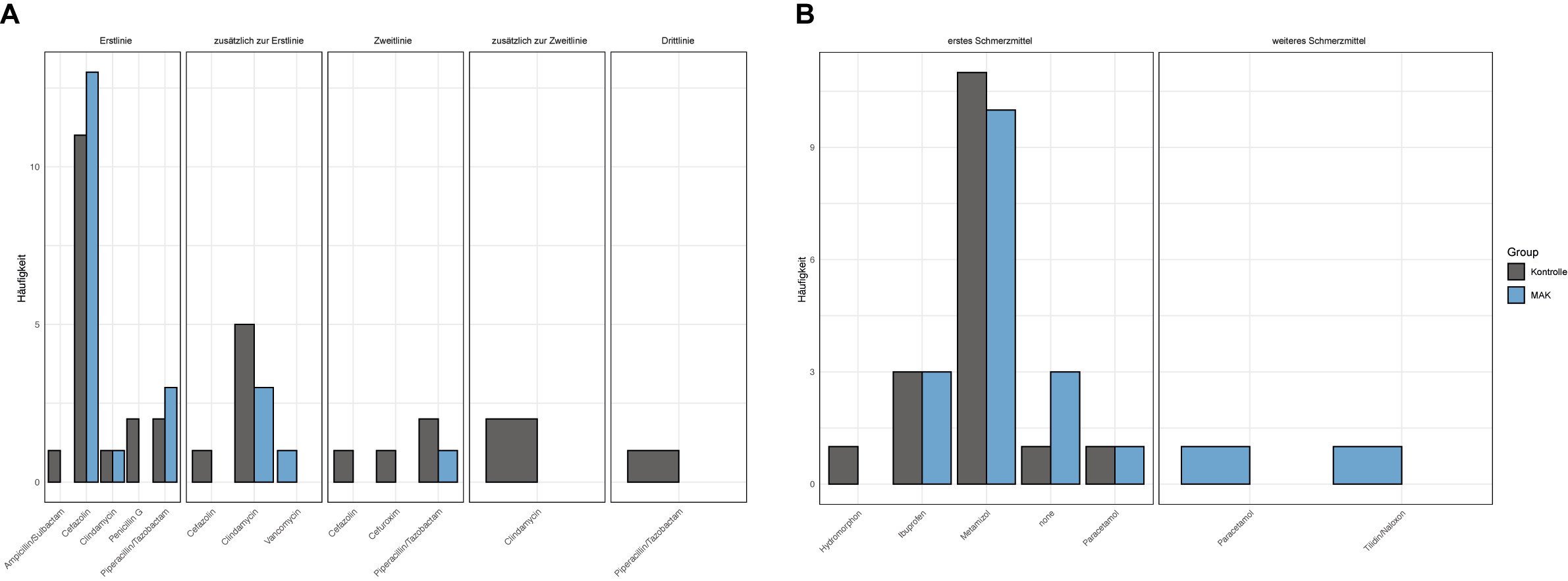

Supplement: Supplementary file 1 — Supplementary information [file DDG-23-1274-s001.jpg]
